# Gun-Shot Wounds of the Jaw and Facial Injuries

**Published:** 1920-04-03

**Authors:** 


### GUN-SHOT WOUNDS OF THE JAW AND FACIAL INJURIES.

### I. Some Nursing Aspects.

[The following very brief description of some nursing points in connection with the injury described is the result of personal experience when working under the direction of Major V. H. Kazanjian, C.M.G., of the Harvard Surgical Unit, attached R.A.M.C., B.E.F., in France, at a hospital at Camiers during the recent war, and from notes taken from a supplement of the *British Dental Journal*.]

The gunshot wound of the jaw and its attendant facial injuries became during the recent war a subject of much importance in the hospital world and opened up a new branch of study in the nursing aspects relating to it and to oral surgery. Prior to the war the jaw injury was seldom met with in civil hospital practice, and then only under conditions having uniform symptoms and presenting appearances very similar in every case. But the shattered jaw and face, together with the possible destruction of four out of the five cardinal senses, was a type of injury which required special treatment and specialist knowledge. Innumerable injuries of this type were inflicted owing to the exposure in modern trench warfare of the head and face. These cases in the early days were admitted as general casualties into the ordinary wards of the hospitals under no other classification except that of G.S.W., and they speedily assumed the characteristics of a highly septic and serious type of war injury.

So many are the disabilities of such patients, and so great their urgent and constant needs, that these compel the need of specialisation and separate treatment in buildings equipped for the purpose with every requirement necessary for dental work. Prompt and efficient treatment for all jaw injuries is of the first importance. Immediate treatment holds the hope of securing the best possible permanent results and materially assists the future work of restoring the functions of the jaw, the powers of mastication and speech, and, finally, the appearance of the face. Delayed, neglected treatment is certain to produce deformities and malunions which are hopeless of correction and inevitably condemn the unfortunate patient to an unenviable existence.

The treatment is a constructive process of slow stages rather out of the ordinary surgical sphere and coming partly within that of the dental, dependent upon the co-operation of both for the best results. The experience that specialisation has afforded has made for considerable progress in the clinical and nursing aspects of jaw injuries. The intelligent co-operation of the nurse becomes a primary factor in following the technical work throughout its varying intricacies. Inadvertent or careless handling may so easily result in misplacing a delicate contour or union. The correct adjustment of the splints has to be clearly understood in relation to the dressing and cleansing of the mouth and wounds and also the feeding of the patient. Every case presents distinctions and peculiarities, which have to be studied and treated accordingly. The nursing aspects open out a wide field of practical and valuable experience requiring from the nurse individual skill, consistent conscientious care, comprehension, and resource. Her work concerns not only the restoration of health or of a living countenance comely and seemly to look upon; in close combination with these duties is the healing of the mind toward a normal outlook and regaining a normal grip in the struggle for existence.

### Description of Injury.

Even though the injury be extensive and severe, consciousness is not always lost at the time, and men so injured have been known to care for themselves in a most marvellous way, even to controlling hæmorrhage. The extent of the injury greatly varies and cannot always be estimated from the external aspect. A bullet may leave very small entry and exit wounds, but may have done irreparable mischief in the course of its passage, as, for instance, behind the angle of the jaw or in the neighbourhood of the neck. The injury may be very slight, involving only the teeth and neighbouring parts without damage to the bone; on the other hand, there may be multiple comminuted fractures of the jaw and small bones of the face and the teeth, with portions of the jaw impacted. Within the mouth there may be laceration and contusion of soft tissues and the tongue; externally an area of wound varying in extent, which may include devastation of the eyes, nose, ears, tongue, and pharynx. The exposed tissues and torn fragments rapidly become inflamed, septic, or gangrenous; toxic absorption in turn creates constitutional disturbances and disorders in the alimentary system. The discharges of the mouth are purulent and fœtid; salivation is excessive and uncontrollable, proving a source of complication to the satisfactory progress of treatment and an additional discomfort and annoyance to the patient. There may be other more or less severe wounds to other parts of the body. The patient suffers greatly from shock; even though slightly wounded, signs of extreme weakness and fatigue are plainly shown and persist for a lengthy period of time.

Such a sufferer presents the most pitiful example of an injury due to modern warfare. He is deprived of speech to express his suffering, possibly of both sight and hearing and the means of conveying nourishment into the body. Upon such a condition inevitably follows a profound mental depression, a lowered vitality and power of resistance due in a large measure to the effects of semi-starvation.

### Initial Stages: Treatment.

The physical condition of the patient demands the first consideration. The presence of other injuries or maladies is ascertained as to their extent and severity. These may be of such a nature as to compel attention before the facial condition. Every effort must be directed toward the state of shock and collapse by the prompt administration of warmth, nourishment, and stimulants (if prescribed), and by securing sleep to the patient. The necessity of food is usually of first importance, and before the fatigue of the toilet is undertaken a meal light and nourishing in character should be given. Owing to his obvious disability the patient may have been without sustenance for some time previously, and until his state of semi-starvation is remedied his condition is grave and his sufferings are intensified. Where signs of hæmorrhage are indicated by a blanched anæmic appearance, stimulants if given must be cautiously administered, as the combined effects of heat, food and stimulants upon a rising blood pressure may give rise to a recurrence. Not until all signs of

**Gunshot Wounds of the Jaw**—(*continued*).

extreme weakness have disappeared can any but superficial cleansing be given to the facial condition. Hypnotics may be necessary to relieve pain and secure sleep, or to quieten delirium where much fever is present. The lines of general health should be established and the elimination of toxic products by a discreet use of purgatives.

Oral Treatment.

As soon as the physical condition of the patient is sufficiently restored a thorough investigation should be made of the injury affecting the jaws, the face, and all surrounding parts; the extent of the fractures and location of foreign bodies is to be confirmed by *x*-ray photographs. Most useful as records are camera portraits taken from various angles at this stage and casts of the face made in plaster. The patient in bed is best kept in the sitting position, supported by a bed-rest and plenty of pillows. This allows for greater comfort and ease in breathing, provides for the outward drainage of all the foul secretions of the mouth, and greatly facilitates the bedside work of the doctor and nurse; it also militates against chest troubles. The interior of the mouth will be found in an offensive condition of suppuration and sloughing lacerated tissue. The cavity should be thoroughly explored with mouth mirror and curved forceps, all irritating and infective material removed, and the mouth cleansed of all debris that can be detached without force. A procedure commonly used and found to be very beneficial in maintaining the cleanliness of the mouth and parts was as follows:

1. Irrigation with a solution of Condy's Fluid.
2. Tincture of iodine on cottonwool, applied to the gums and teeth and unbroken surfaces.
3. Peroxide of hydrogen on cottonwool to the mucous surfaces and teeth.
4. Irrigation of the mouth with an antiseptic. Listerine is useful for its cleansing properties and pleasant flavour.

This treatment is repeated as often as possible; every two hours, if it can be borne by the patient, for the first twenty-four hours at least. The importance of consistent and frequent dressing will be appreciated and understood by the ever-present possibility of complications which compel the serious and urgent cleansing of the mouth. The relief and comfort that is experienced is such that repeated dressing is welcome, and is followed within the first twenty-four hours by marked improvement; afterwards the treatment can be continued every three or four hours, or arranged before and after each meal. Tincture of iodine is applied only once a day. The excessive salivation is an annoyance, and a source of harm in that it provides a medium of contamination, conveying infective agents and pus to the wounds, and, by being swallowed, to the stomach. Means for its outward flow must be devised and provided, and a plentiful supply of soft old linen for use as handkerchiefs, which should be afterwards burnt. In contrast is the predisposition to excessive dryness of the mouth, where the secretory glands have been destroyed, or a deformity of the jaws exists which prevents the closing of the mouth. The mucous surfaces are exposed to the atmosphere, and, lacking the normal lubricant of the salivary glands, become dry and cracked. This condition is equally distressing; to moisten the surfaces, spray frequently with a mixture composed of:

| | | |
|---|---|---|
| R. | Ol. menth. pip. | ♏ss. |
| | Liq. cresolis co. | ♏ii. |
| | Paraffin liquid | ʒj. |

(*To be continued.*)

